# Supplementary material for: Trends in skin cancer incidence in Songkhla, Southern Thailand, 1989–2020: A population-based study on the impact of geographic variation
Source: PLoS One. 2026 Jan 20;21(1):e0331635. doi: 10.1371/journal.pone.0331635 (PMC12818597; doi:10.1371/journal.pone.0331635)
Supplement: S1 Table — (DOCX) [file pone.0331635.s001.docx]

**S1 table.** Trends in skin cancer incidence by sex in Songkhla, Thailand, from 1989 to 2020, based on the Joinpoint regression analysis

| Sex | Men | | | | Women | | | |
| --- | --- | --- | --- | --- | --- | --- | --- | --- |
| Year | ASR | Model ASR | Standard Error | APCC (95% CI) | ASR | Model ASR | Standard Error | APCC (95% CI) |
| 1989 | 2.37 | 2.90 | 0.77 | 5.55 (0.67, 90.50) | 3.01 | 3.53 | 0.86 | 0.24 (-0.68, 1.17) |
| 1990 | 2.27 | 3.06 | 0.73 | 5.55 (0.67, 90.50) | 2.01 | 3.54 | 0.65 | 0.24 (-0.68, 1.17) |
| 1991 | 3.55 | 3.23 | 0.96 | 5.55 (0.67, 90.50) | 3.72 | 3.55 | 0.92 | 0.24 (-0.68, 1.17) |
| 1992 | 2.02 | 3.41 | 0.68 | 5.55 (0.67, 90.50) | 4.04 | 3.56 | 0.90 | 0.24 (-0.68, 1.17) |
| 1993 | 4.88 | 3.60 | 1.08 | 5.55 (0.67, 90.50) | 3.72 | 3.57 | 0.85 | 0.24 (-0.68, 1.17) |
| 1994 | 4.99 | 3.80 | 1.08 | 5.55 (0.67, 90.50) | 2.6 | 3.58 | 0.68 | 0.24 (-0.68, 1.17) |
| 1995 | 5.11 | 4.01 | 1.06 | 5.55 (0.67, 90.50) | 3.45 | 3.59 | 0.79 | 0.24 (-0.68, 1.17) |
| 1996 | 3.36 | 4.24 | 0.85 | 5.55 (0.67, 90.50) | 5.94 | 3.59 | 1.05 | 0.24 (-0.68, 1.17) |
| 1997 | 2.69 | 4.47 | 0.75 | 5.55 (0.67, 90.50) | 2.65 | 3.60 | 0.66 | 0.24 (-0.68, 1.17) |
| 1998 | 5.12 | 4.72 | 0.99 | 5.55 (0.67, 90.50) | 3.74 | 3.61 | 0.77 | 0.24 (-0.68, 1.17) |
| 1999 | 4.72 | 4.98 | 0.95 | 5.55 (0.67, 90.50) | 2.45 | 3.62 | 0.62 | 0.24 (-0.68, 1.17) |
| 2000 | 5.27 | 5.26 | 1.00 | 5.55 (0.67, 90.50) | 4.59 | 3.63 | 0.84 | 0.24 (-0.68, 1.17) |
| 2001 | 6.42 | 5.55 | 1.08 | joinpoint | 3.93 | 3.64 | 0.80 | 0.24 (-0.68, 1.17) |
| 2002 | 4.53 | 5.43 | 0.90 | -2.24 (-13.65, -0.42) | 3.64 | 3.65 | 0.74 | 0.24 (-0.68, 1.17) |
| 2003 | 4.23 | 5.3 | 0.83 | -2.24 (-13.65, -0.42) | 3.69 | 3.66 | 0.73 | 0.24 (-0.68, 1.17) |
| 2004 | 5.39 | 5.19 | 0.91 | -2.24 (-13.65, -0.42) | 3.92 | 3.66 | 0.75 | 0.24 (-0.68, 1.17) |
| 2005 | 4.62 | 5.07 | 0.84 | -2.24 (-13.65, -0.42) | 2.35 | 3.67 | 0.53 | 0.24 (-0.68, 1.17) |
| 2006 | 3.06 | 4.96 | 0.67 | -2.24 (-13.65, -0.42) | 3.93 | 3.68 | 0.68 | 0.24 (-0.68, 1.17) |
| 2007 | 4.41 | 4.85 | 0.78 | -2.24 (-13.65, -0.42) | 3.08 | 3.69 | 0.62 | 0.24 (-0.68, 1.17) |
| 2008 | 4.97 | 4.74 | 0.85 | -2.24 (-13.65, -0.42) | 3.65 | 3.70 | 0.64 | 0.24 (-0.68, 1.17) |
| 2009 | 6.00 | 4.63 | 0.89 | -2.24 (-13.65, -0.42) | 3.52 | 3.71 | 0.64 | 0.24 (-0.68, 1.17) |
| 2010 | 5.26 | 4.53 | 0.83 | -2.24 (-13.65, -0.42) | 3.17 | 3.72 | 0.58 | 0.24 (-0.68, 1.17) |
| 2011 | 5.38 | 4.43 | 0.83 | -2.24 (-13.65, -0.42) | 3.91 | 3.73 | 0.63 | 0.24 (-0.68, 1.17) |
| 2012 | 4.49 | 4.33 | 0.77 | -2.24 (-13.65, -0.42) | 3.15 | 3.74 | 0.57 | 0.24 (-0.68, 1.17) |
| 2013 | 3.79 | 4.23 | 0.68 | -2.24 (-13.65, -0.42) | 3.44 | 3.74 | 0.59 | 0.24 (-0.68, 1.17) |
| 2014 | 3.03 | 4.14 | 0.59 | -2.24 (-13.65, -0.42) | 4.06 | 3.75 | 0.61 | 0.24 (-0.68, 1.17) |
| 2015 | 4.42 | 4.04 | 0.70 | -2.24 (-13.65, -0.42) | 4.68 | 3.76 | 0.66 | 0.24 (-0.68, 1.17) |
| 2016 | 4.61 | 3.95 | 0.70 | -2.24 (-13.65, -0.42) | 5.02 | 3.77 | 0.67 | 0.24 (-0.68, 1.17) |
| 2017 | 4.84 | 3.86 | 0.71 | -2.24 (-13.65, -0.42) | 4.55 | 3.78 | 0.63 | 0.24 (-0.68, 1.17) |
| 2018 | 3.05 | 3.78 | 0.56 | -2.24 (-13.65, -0.42) | 3.58 | 3.79 | 0.56 | 0.24 (-0.68, 1.17) |
| 2019 | 3.35 | 3.69 | 0.58 | -2.24 (-13.65, -0.42) | 2.82 | 3.80 | 0.48 | 0.24 (-0.68, 1.17) |
| 2020 | 2.71 | 3.61 | 0.49 | -2.24 (-13.65, -0.42) | 2.67 | 3.81 | 0.47 | 0.24 (-0.68, 1.17) |
