# Supplementary material for: Trends in skin cancer incidence in Songkhla, Southern Thailand, 1989–2020: A population-based study on the impact of geographic variation
Source: PLoS One. 2026 Jan 20;21(1):e0331635. doi: 10.1371/journal.pone.0331635 (PMC12818597; doi:10.1371/journal.pone.0331635)
Supplement: S2 Table — (DOCX) [file pone.0331635.s002.docx]

**S2 table.** Trends in skin cancer incidence by geographic areas in Songkhla, Thailand, from 1989 to 2020, based on the Joinpoint regression analysis

| Sex | | Men | | | | Women | | | |
| --- | --- | --- | --- | --- | --- | --- | --- | --- | --- |
| Area | Year | ASR | Model ASR | Standard Error | APCC (95%CI) | ASR | Model ASR | Standard Error | APCC (95%CI) |
| City | 1997 | 4.01 | 7.77 | 1.66 | -1.07 (-2.72, 0.90) | 3.21 | 4.72 | 1.26 | 0.22 (-1.14, 1.59) |
| City | 1998 | 8.01 | 7.69 | 2.26 | -1.07 (-2.72, 0.90) | 5.25 | 4.73 | 1.55 | 0.22 (-1.14, 1.59) |
| City | 1999 | 8.98 | 7.61 | 2.43 | -1.07 (-2.72, 0.90) | 3.66 | 4.74 | 1.31 | 0.22 (-1.14, 1.59) |
| City | 2000 | 5.45 | 7.53 | 1.83 | -1.07 (-2.72, 0.90) | 5.20 | 4.75 | 1.53 | 0.22 (-1.14, 1.59) |
| City | 2001 | 8.29 | 7.45 | 2.18 | -1.07 (-2.72, 0.90) | 5.10 | 4.76 | 1.57 | 0.22 (-1.14, 1.59) |
| City | 2002 | 6.35 | 7.37 | 1.87 | -1.07 (-2.72, 0.90) | 6.29 | 4.77 | 1.65 | 0.22 (-1.14, 1.59) |
| City | 2003 | 7.11 | 7.29 | 1.94 | -1.07 (-2.72, 0.90) | 6.17 | 4.78 | 1.68 | 0.22 (-1.14, 1.59) |
| City | 2004 | 8.06 | 7.21 | 1.93 | -1.07 (-2.72, 0.90) | 4.33 | 4.79 | 1.34 | 0.22 (-1.14, 1.59) |
| City | 2005 | 7.13 | 7.13 | 1.88 | -1.07 (-2.72, 0.90) | 2.97 | 4.80 | 1.02 | 0.22 (-1.14, 1.59) |
| City | 2006 | 5.88 | 7.06 | 1.65 | -1.07 (-2.72, 0.90) | 4.36 | 4.81 | 1.22 | 0.22 (-1.14, 1.59) |
| City | 2007 | 6.81 | 6.98 | 1.79 | -1.07 (-2.72, 0.90) | 3.98 | 4.82 | 1.19 | 0.22 (-1.14, 1.59) |
| City | 2008 | 6.41 | 6.91 | 1.64 | -1.07 (-2.72, 0.90) | 4.60 | 4.83 | 1.15 | 0.22 (-1.14, 1.59) |
| City | 2009 | 10.12 | 6.83 | 2.02 | -1.07 (-2.72, 0.90) | 5.69 | 4.85 | 1.35 | 0.22 (-1.14, 1.59) |
| City | 2010 | 5.62 | 6.76 | 1.53 | -1.07 (-2.72, 0.90) | 4.15 | 4.86 | 1.08 | 0.22 (-1.14, 1.59) |
| City | 2011 | 8.36 | 6.69 | 1.85 | -1.07 (-2.72, 0.90) | 4.63 | 4.87 | 1.12 | 0.22 (-1.14, 1.59) |
| City | 2012 | 7.08 | 6.62 | 1.65 | -1.07 (-2.72, 0.90) | 3.98 | 4.88 | 1.07 | 0.22 (-1.14, 1.59) |
| City | 2013 | 4.59 | 6.55 | 1.26 | -1.07 (-2.72, 0.90) | 5.43 | 4.89 | 1.32 | 0.22 (-1.14, 1.59) |
| City | 2014 | 4.74 | 6.48 | 1.25 | -1.07 (-2.72, 0.90) | 4.76 | 4.90 | 1.08 | 0.22 (-1.14, 1.59) |
| City | 2015 | 5.64 | 6.41 | 1.31 | -1.07 (-2.72, 0.90) | 5.91 | 4.91 | 1.22 | 0.22 (-1.14, 1.59) |
| City | 2016 | 8.95 | 6.34 | 1.64 | -1.07 (-2.72, 0.90) | 5.59 | 4.92 | 1.11 | 0.22 (-1.14, 1.59) |
| City | 2017 | 8.01 | 6.27 | 1.51 | -1.07 (-2.72, 0.90) | 6.49 | 4.93 | 1.23 | 0.22 (-1.14, 1.59) |
| City | 2018 | 5.49 | 6.21 | 1.28 | -1.07 (-2.72, 0.90) | 5.09 | 4.94 | 1.06 | 0.22 (-1.14, 1.59) |
| City | 2019 | 4.33 | 6.14 | 1.10 | -1.07 (-2.72, 0.90) | 3.89 | 4.95 | 0.90 | 0.22 (-1.14, 1.59) |
| City | 2020 | 4.77 | 6.07 | 1.08 | -1.07 (-2.72, 0.90) | 3.20 | 4.96 | 0.86 | 0.22 (-1.14, 1.59) |
| Rural | 1997 | 2.54 | 3.92 | 0.96 | 1.84 (-1.76, 56.06) | 2.58 | 2.90 | 0.85 | 1.93* (0.33, 30.08) |
| Rural | 1998 | 4.24 | 3.99 | 1.15 | 1.84 (-1.76, 56.06) | 3.26 | 2.95 | 0.96 | 1.93* (0.33, 30.08) |
| Rural | 1999 | 3.15 | 4.06 | 0.97 | 1.84 (-1.76, 56.06) | 1.94 | 3.01 | 0.72 | 1.93* (0.33, 30.08) |
| Rural | 2000 | 5.67 | 4.14 | 1.32 | 1.84 (-1.76, 56.06) | 4.82 | 3.07 | 1.14 | 1.93* (0.33, 30.08) |
| Rural | 2001 | 6.12 | 4.21 | 1.36 | 1.84 (-1.76, 56.06) | 3.57 | 3.13 | 0.98 | 1.93* (0.33, 30.08) |
| Rural | 2002 | 4.19 | 4.29 | 1.13 | 1.84 (-1.76, 56.06) | 2.44 | 3.19 | 0.78 | 1.93* (0.33, 30.08) |
| Rural | 2003 | 3.25 | 4.37 | 0.95 | 1.84 (-1.76, 56.06) | 2.67 | 3.25 | 0.78 | 1.93* (0.33, 30.08) |
| Rural | 2004 | 4.39 | 4.45 | 1.05 | 1.84 (-1.76, 56.06) | 3.92 | 3.31 | 0.95 | 1.93* (0.33, 30.08) |
| Rural | 2005 | 4.04 | 4.53 | 1.03 | 1.84 (-1.76, 56.06) | 2.54 | 3.38 | 0.76 | 1.93* (0.33, 30.08) |
| Rural | 2006 | 2.24 | 4.61 | 0.78 | 1.84 (-1.76, 56.06) | 4.02 | 3.44 | 0.90 | 1.93* (0.33, 30.08) |
| Rural | 2007 | 4.00 | 4.70 | 0.98 | 1.84 (-1.76, 56.06) | 3.08 | 3.51 | 0.84 | 1.93* (0.33, 30.08) |
| Rural | 2008 | 5.04 | 4.79 | 1.15 | 1.84 (-1.76, 56.06) | 3.60 | 3.57 | 0.87 | 1.93* (0.33, 30.08) |
| Rural | 2009 | 4.75 | 4.87 | 1.07 | 1.84 (-1.76, 56.06) | 2.78 | 3.64 | 0.79 | 1.93* (0.33, 30.08) |
| Rural | 2010 | 6.30 | 4.96 | 1.21 | joinpoint | 3.29 | 3.71 | 0.83 | 1.93* (0.33, 30.08) |
| Rural | 2011 | 4.63 | 4.60 | 1.01 | -7.40* (-40.91, -2.45) | 4.19 | 3.79 | 0.90 | 1.93* (0.33, 30.08) |
| Rural | 2012 | 3.90 | 4.26 | 0.99 | -7.40* (-40.91, -2.45) | 3.18 | 3.86 | 0.77 | 1.93* (0.33, 30.08) |
| Rural | 2013 | 3.99 | 3.94 | 0.96 | -7.40* (-40.91, -2.45) | 2.76 | 3.93 | 0.68 | 1.93* (0.33, 30.08) |
| Rural | 2014 | 2.46 | 3.65 | 0.71 | -7.40* (-40.91, -2.45) | 4.06 | 4.01 | 0.84 | 1.93* (0.33, 30.08) |
| Rural | 2015 | 4.22 | 3.38 | 0.92 | -7.40* (-40.91, -2.45) | 4.51 | 4.09 | 0.89 | 1.93* (0.33, 30.08) |
| Rural | 2016 | 2.68 | 3.13 | 0.75 | -7.40* (-40.91, -2.45) | 5.23 | 4.17 | 0.94 | joinpoint |
| Rural | 2017 | 3.76 | 2.9 | 0.89 | -7.40* (-40.91, -2.45) | 3.70 | 3.64 | 0.76 | -12.72 (-37.85, 0.02) |
| Rural | 2018 | 1.96 | 2.68 | 0.59 | -7.40* (-40.91, -2.45) | 3.01 | 3.17 | 0.73 | -12.72 (-37.85, 0.02) |
| Rural | 2019 | 3.12 | 2.48 | 0.77 | -7.40* (-40.91, -2.45) | 2.44 | 2.77 | 0.61 | -12.72 (-37.85, 0.02) |
| Rural | 2020 | 1.74 | 2.30 | 0.54 | -7.40* (-40.91, -2.45) | 2.69 | 2.42 | 0.64 | -12.72 (-37.85, 0.02) |
| Fishing | 1997 | 6.43 | 6.44 | 2.87 | -1.71 (-5.07, 1.74) | 4.61 | 3.11 | 2.10 | 2.63 (-0.08, 37085) |
| Fishing | 1998 | 3.87 | 6.33 | 1.97 | -1.71 (-5.07, 1.74) | 2.02 | 3.19 | 1.20 | 2.63 (-0.08, 37085) |
| Fishing | 1999 | 2.01 | 6.22 | 1.42 | -1.71 (-5.07, 1.74) | 0.53 | 3.27 | 0.53 | 2.63 (-0.08, 37085) |
| Fishing | 2000 | 4.50 | 6.11 | 2.04 | -1.71 (-5.07, 1.74) | 3.98 | 3.36 | 1.83 | 2.63 (-0.08, 37085) |
| Fishing | 2001 | 9.70 | 6.01 | 2.97 | -1.71 (-5.07, 1.74) | 4.47 | 3.45 | 1.86 | 2.63 (-0.08, 37085) |
| Fishing | 2002 | 8.54 | 5.91 | 2.89 | -1.71 (-5.07, 1.74) | 2.22 | 3.54 | 1.29 | 2.63 (-0.08, 37085) |
| Fishing | 2003 | 4.96 | 5.81 | 2.23 | -1.71 (-5.07, 1.74) | 3.98 | 3.63 | 1.71 | 2.63 (-0.08, 37085) |
| Fishing | 2004 | 3.04 | 5.71 | 1.54 | -1.71 (-5.07, 1.74) | 6.21 | 3.73 | 2.03 | 2.63 (-0.08, 37085) |
| Fishing | 2005 | 4.99 | 5.61 | 1.97 | -1.71 (-5.07, 1.74) | 2.58 | 3.83 | 1.37 | 2.63 (-0.08, 37085) |
| Fishing | 2006 | 3.05 | 5.51 | 1.62 | -1.71 (-5.07, 1.74) | 3.08 | 3.93 | 1.22 | 2.63 (-0.08, 37085) |
| Fishing | 2007 | 4.77 | 5.42 | 1.92 | -1.71 (-5.07, 1.74) | 4.16 | 4.03 | 1.76 | 2.63 (-0.08, 37085) |
| Fishing | 2008 | 5.17 | 5.33 | 2.15 | -1.71 (-5.07, 1.74) | 3.76 | 4.14 | 1.50 | 2.63 (-0.08, 37085) |
| Fishing | 2009 | 5.82 | 5.23 | 2.03 | -1.71 (-5.07, 1.74) | 3.07 | 4.24 | 1.34 | 2.63 (-0.08, 37085) |
| Fishing | 2010 | 9.07 | 5.15 | 2.68 | -1.71 (-5.07, 1.74) | 2.11 | 4.36 | 1.13 | 2.63 (-0.08, 37085) |
| Fishing | 2011 | 5.03 | 5.06 | 1.96 | -1.71 (-5.07, 1.74) | 5.24 | 4.47 | 1.75 | 2.63 (-0.08, 37085) |
| Fishing | 2012 | 5.66 | 4.97 | 2.19 | -1.71 (-5.07, 1.74) | 3.48 | 4.59 | 1.47 | 2.63 (-0.08, 37085) |
| Fishing | 2013 | 3.76 | 4.89 | 1.64 | -1.71 (-5.07, 1.74) | 3.00 | 4.71 | 1.31 | 2.63 (-0.08, 37085) |
| Fishing | 2014 | 3.57 | 4.80 | 1.66 | -1.71 (-5.07, 1.74) | 6.44 | 4.83 | 2.02 | 2.63 (-0.08, 37085) |
| Fishing | 2015 | 6.05 | 4.72 | 2.10 | -1.71 (-5.07, 1.74) | 4.94 | 4.96 | 1.58 | 2.63 (-0.08, 37085) |
| Fishing | 2016 | 1.96 | 4.64 | 1.16 | -1.71 (-5.07, 1.74) | 5.78 | 5.09 | 1.78 | joinpoint |
| Fishing | 2017 | 6.66 | 4.56 | 2.29 | -1.71 (-5.07, 1.74) | 4.77 | 3.87 | 1.56 | -23.95* (-69.52, -0.90) |
| Fishing | 2018 | 2.10 | 4.48 | 1.11 | -1.71 (-5.07, 1.74) | 1.65 | 2.94 | 1.00 | -23.95* (-69.52, -0.90) |
| Fishing | 2019 | 5.48 | 4.41 | 1.91 | -1.71 (-5.07, 1.74) | 2.65 | 2.24 | 1.17 | -23.95* (-69.52, -0.90) |
| Fishing | 2020 | 1.80 | 4.33 | 0.98 | -1.71 (-5.07, 1.74) | 1.56 | 1.70 | 0.78 | -23.95* (-69.52, -0.90) |
| Non-fishing | 1997 | 2.17 | 5.88 | 0.77 | -1.45 (-3.13, 0.44) | 2.25 | 3.50 | 0.67 | 1.35* (0.18, 20.54) |
| Non-fishing | 1998 | 5.93 | 5.79 | 1.25 | -1.45 (-3.13, 0.44) | 4.50 | 3.55 | 1.01 | 1.35* (0.18, 20.54) |
| Non-fishing | 1999 | 5.8 | 5.71 | 1.22 | -1.45 (-3.13, 0.44) | 3.03 | 3.59 | 0.80 | 1.35* (0.18, 20.54) |
| Non-fishing | 2000 | 5.97 | 5.63 | 1.26 | -1.45 (-3.13, 0.44) | 5.09 | 3.64 | 1.03 | 1.35* (0.18, 20.54) |
| Non-fishing | 2001 | 6.00 | 5.55 | 1.21 | -1.45 (-3.13, 0.44) | 4.03 | 3.69 | 0.95 | 1.35* (0.18, 20.54) |
| Non-fishing | 2002 | 4.00 | 5.47 | 0.98 | -1.45 (-3.13, 0.44) | 4.20 | 3.74 | 0.91 | 1.35* (0.18, 20.54) |
| Non-fishing | 2003 | 4.56 | 5.39 | 1.01 | -1.45 (-3.13, 0.44) | 3.90 | 3.79 | 0.88 | 1.35* (0.18, 20.54) |
| Non-fishing | 2004 | 6.35 | 5.31 | 1.14 | -1.45 (-3.13, 0.44) | 3.60 | 3.84 | 0.85 | 1.35* (0.18, 20.54) |
| Non-fishing | 2005 | 5.21 | 5.23 | 1.08 | -1.45 (-3.13, 0.44) | 2.68 | 3.90 | 0.67 | 1.35* (0.18, 20.54) |
| Non-fishing | 2006 | 3.51 | 5.16 | 0.85 | -1.45 (-3.13, 0.44) | 4.37 | 3.95 | 0.84 | 1.35* (0.18, 20.54) |
| Non-fishing | 2007 | 4.90 | 5.08 | 0.99 | -1.45 (-3.13, 0.44) | 3.28 | 4.00 | 0.76 | 1.35* (0.18, 20.54) |
| Non-fishing | 2008 | 5.65 | 5.01 | 1.06 | -1.45 (-3.13, 0.44) | 4.01 | 4.06 | 0.78 | 1.35* (0.18, 20.54) |
| Non-fishing | 2009 | 6.97 | 4.93 | 1.15 | -1.45 (-3.13, 0.44) | 4.18 | 4.11 | 0.84 | 1.35* (0.18, 20.54) |
| Non-fishing | 2010 | 5.26 | 4.86 | 0.98 | -1.45 (-3.13, 0.44) | 3.86 | 4.17 | 0.75 | 1.35* (0.18, 20.54) |
| Non-fishing | 2011 | 6.25 | 4.79 | 1.06 | -1.45 (-3.13, 0.44) | 4.03 | 4.22 | 0.74 | 1.35* (0.18, 20.54) |
| Non-fishing | 2012 | 4.78 | 4.72 | 0.92 | -1.45 (-3.13, 0.44) | 3.42 | 4.28 | 0.69 | 1.35* (0.18, 20.54) |
| Non-fishing | 2013 | 4.26 | 4.65 | 0.85 | -1.45 (-3.13, 0.44) | 3.82 | 4.34 | 0.71 | 1.35* (0.18, 20.54) |
| Non-fishing | 2014 | 3.26 | 4.59 | 0.69 | -1.45 (-3.13, 0.44) | 3.86 | 4.4 | 0.67 | 1.35* (0.18, 20.54) |
| Non-fishing | 2015 | 4.43 | 4.52 | 0.80 | -1.45 (-3.13, 0.44) | 5.02 | 4.46 | 0.80 | 1.35* (0.18, 20.54) |
| Non-fishing | 2016 | 5.75 | 4.45 | 0.91 | -1.45 (-3.13, 0.44) | 5.30 | 4.52 | 0.79 | 1.35* (0.18, 20.54) |
| Non-fishing | 2017 | 5.14 | 4.39 | 0.84 | -1.45 (-3.13, 0.44) | 4.79 | 4.58 | 0.74 | joinpoint |
| Non-fishing | 2018 | 3.6 | 4.33 | 0.71 | -1.45 (-3.13, 0.44) | 4.31 | 3.97 | 0.71 | -13.20 (-34.05, 0.23) |
| Non-fishing | 2019 | 3.19 | 4.26 | 0.66 | -1.45 (-3.13, 0.44) | 3.13 | 3.45 | 0.58 | -13.20 (-34.05, 0.23) |
| Non-fishing | 2020 | 3.13 | 4.20 | 0.61 | -1.45 (-3.13, 0.44) | 3.09 | 2.99 | 0.59 | -13.20 (-34.05, 0.23) |
| Rubber | 1997 | - | - | - | - | 1.28 | 3.01 | 0.74 | 1.20 (-1.13, 4.17) |
| Rubber | 1998 | 5.30 | 5.65 | 2.04 | -2.92 (-6.54, 0.82) | 4.11 | 3.05 | 1.70 | 1.20 (-1.13, 4.17) |
| Rubber | 1999 | 4.90 | 5.48 | 1.88 | -2.92 (-6.54, 0.82) | 3.59 | 3.09 | 1.62 | 1.20 (-1.13, 4.17) |
| Rubber | 2000 | 6.24 | 5.32 | 2.22 | -2.92 (-6.54, 0.82) | 4.27 | 3.12 | 1.66 | 1.20 (-1.13, 4.17) |
| Rubber | 2001 | 3.61 | 5.17 | 1.63 | -2.92 (-6.54, 0.82) | 3.98 | 3.16 | 1.65 | 1.20 (-1.13, 4.17) |
| Rubber | 2002 | 3.76 | 5.01 | 1.70 | -2.92 (-6.54, 0.82) | 2.70 | 3.20 | 1.22 | 1.20 (-1.13, 4.17) |
| Rubber | 2003 | 2.44 | 4.87 | 1.24 | -2.92 (-6.54, 0.82) | 1.76 | 3.24 | 0.89 | 1.20 (-1.13, 4.17) |
| Rubber | 2004 | 6.34 | 4.73 | 1.95 | -2.92 (-6.54, 0.82) | 2.81 | 3.28 | 1.27 | 1.20 (-1.13, 4.17) |
| Rubber | 2005 | 2.42 | 4.59 | 1.22 | -2.92 (-6.54, 0.82) | 2.02 | 3.32 | 1.02 | 1.20 (-1.13, 4.17) |
| Rubber | 2006 | 1.03 | 4.45 | 0.73 | -2.92 (-6.54, 0.82) | 4.49 | 3.36 | 1.56 | 1.20 (-1.13, 4.17) |
| Rubber | 2007 | 3.34 | 4.32 | 1.28 | -2.92 (-6.54, 0.82) | 3.37 | 3.40 | 1.41 | 1.20 (-1.13, 4.17) |
| Rubber | 2008 | 7.34 | 4.2 | 2.17 | -2.92 (-6.54, 0.82) | 3.07 | 3.44 | 1.20 | 1.20 (-1.13, 4.17) |
| Rubber | 2009 | 4.94 | 4.07 | 1.80 | -2.92 (-6.54, 0.82) | 3.20 | 3.48 | 1.38 | 1.20 (-1.13, 4.17) |
| Rubber | 2010 | 6.16 | 3.95 | 1.83 | -2.92 (-6.54, 0.82) | 3.48 | 3.52 | 1.23 | 1.20 (-1.13, 4.17) |
| Rubber | 2011 | 5.14 | 3.84 | 1.57 | -2.92 (-6.54, 0.82) | 3.98 | 3.56 | 1.45 | 1.20 (-1.13, 4.17) |
| Rubber | 2012 | 3.13 | 3.73 | 1.29 | -2.92 (-6.54, 0.82) | 2.19 | 3.60 | 0.94 | 1.20 (-1.13, 4.17) |
| Rubber | 2013 | 4.44 | 3.62 | 1.53 | -2.92 (-6.54, 0.82) | 3.63 | 3.65 | 1.21 | 1.20 (-1.13, 4.17) |
| Rubber | 2014 | 1.83 | 3.51 | 0.83 | -2.92 (-6.54, 0.82) | 3.62 | 3.69 | 1.19 | 1.20 (-1.13, 4.17) |
| Rubber | 2015 | 3.49 | 3.41 | 1.20 | -2.92 (-6.54, 0.82) | 4.86 | 3.73 | 1.45 | 1.20 (-1.13, 4.17) |
| Rubber | 2016 | 2.79 | 3.31 | 1.19 | -2.92 (-6.54, 0.82) | 5.68 | 3.78 | 1.53 | 1.20 (-1.13, 4.17) |
| Rubber | 2017 | 3.93 | 3.21 | 1.41 | -2.92 (-6.54, 0.82) | 2.82 | 3.82 | 0.99 | 1.20 (-1.13, 4.17) |
| Rubber | 2018 | 3.04 | 3.12 | 1.12 | -2.92 (-6.54, 0.82) | 4.81 | 3.87 | 1.35 | 1.20 (-1.13, 4.17) |
| Rubber | 2019 | 2.45 | 3.03 | 0.97 | -2.92 (-6.54, 0.82) | 2.45 | 3.92 | 0.87 | 1.20 (-1.13, 4.17) |
| Rubber | 2020 | 0.96 | 2.94 | 0.59 | -2.92 (-6.54, 0.82) | 3.34 | 3.96 | 1.10 | 1.20 (-1.13, 4.17) |
| Non-rubber | 1997 | 4.15 | 6.28 | 1.16 | -1.34 (-3.00, 0.45) | 3.39 | 3.61 | 0.93 | 1.45* (0.29, 6.13) |
| Non-rubber | 1998 | 5.66 | 6.19 | 1.28 | -1.34 (-3.00, 0.45) | 4.01 | 3.66 | 0.97 | 1.45* (0.29, 6.13) |
| Non-rubber | 1999 | 5.09 | 6.11 | 1.21 | -1.34 (-3.00, 0.45) | 2.12 | 3.72 | 0.66 | 1.45* (0.29, 6.13) |
| Non-rubber | 2000 | 5.41 | 6.03 | 1.22 | -1.34 (-3.00, 0.45) | 5.03 | 3.77 | 1.05 | 1.45* (0.29, 6.13) |
| Non-rubber | 2001 | 8.05 | 5.95 | 1.46 | -1.34 (-3.00, 0.45) | 4.27 | 3.82 | 1.00 | 1.45* (0.29, 6.13) |
| Non-rubber | 2002 | 5.35 | 5.87 | 1.18 | -1.34 (-3.00, 0.45) | 4.23 | 3.88 | 0.96 | 1.45* (0.29, 6.13) |
| Non-rubber | 2003 | 5.36 | 5.79 | 1.16 | -1.34 (-3.00, 0.45) | 4.69 | 3.94 | 1.01 | 1.45* (0.29, 6.13) |
| Non-rubber | 2004 | 5.51 | 5.71 | 1.11 | -1.34 (-3.00, 0.45) | 4.64 | 3.99 | 0.97 | 1.45* (0.29, 6.13) |
| Non-rubber | 2005 | 6.34 | 5.64 | 1.24 | -1.34 (-3.00, 0.45) | 2.81 | 4.05 | 0.70 | 1.45* (0.29, 6.13) |
| Non-rubber | 2006 | 4.34 | 5.56 | 0.99 | -1.34 (-3.00, 0.45) | 4.06 | 4.11 | 0.81 | 1.45* (0.29, 6.13) |
| Non-rubber | 2007 | 5.51 | 5.49 | 1.11 | -1.34 (-3.00, 0.45) | 3.35 | 4.17 | 0.78 | 1.45* (0.29, 6.13) |
| Non-rubber | 2008 | 4.85 | 5.41 | 1.01 | -1.34 (-3.00, 0.45) | 4.29 | 4.23 | 0.84 | 1.45* (0.29, 6.13) |
| Non-rubber | 2009 | 7.52 | 5.34 | 1.22 | -1.34 (-3.00, 0.45) | 4.24 | 4.29 | 0.85 | 1.45* (0.29, 6.13) |
| Non-rubber | 2010 | 5.86 | 5.27 | 1.08 | -1.34 (-3.00, 0.45) | 3.5 | 4.35 | 0.74 | 1.45* (0.29, 6.13) |
| Non-rubber | 2011 | 6.29 | 5.20 | 1.13 | -1.34 (-3.00, 0.45) | 4.43 | 4.42 | 0.79 | 1.45* (0.29, 6.13) |
| Non-rubber | 2012 | 5.69 | 5.13 | 1.08 | -1.34 (-3.00, 0.45) | 3.86 | 4.48 | 0.76 | 1.45* (0.29, 6.13) |
| Non-rubber | 2013 | 4.13 | 5.06 | 0.88 | -1.34 (-3.00, 0.45) | 3.69 | 4.55 | 0.75 | 1.45* (0.29, 6.13) |
| Non-rubber | 2014 | 3.88 | 4.99 | 0.83 | -1.34 (-3.00, 0.45) | 4.54 | 4.61 | 0.78 | 1.45* (0.29, 6.13) |
| Non-rubber | 2015 | 5.09 | 4.92 | 0.92 | -1.34 (-3.00, 0.45) | 5.19 | 4.68 | 0.84 | 1.45* (0.29, 6.13) |
| Non-rubber | 2016 | 5.87 | 4.86 | 0.97 | -1.34 (-3.00, 0.45) | 5.31 | 4.75 | 0.81 | 1.45* (0.29, 6.13) |
| Non-rubber | 2017 | 5.75 | 4.79 | 0.93 | -1.34 (-3.00, 0.45) | 5.45 | 4.82 | 0.83 | joinpoint |
| Non-rubber | 2018 | 3.45 | 4.73 | 0.74 | -1.34 (-3.00, 0.45) | 3.45 | 3.91 | 0.67 | -18.82* (-44.51, -1.68) |
| Non-rubber | 2019 | 3.96 | 4.67 | 0.78 | -1.34 (-3.00, 0.45) | 3.20 | 3.17 | 0.62 | -18.82* (-44.51, -1.68) |
| Non-rubber | 2020 | 3.57 | 4.60 | 0.68 | -1.34 (-3.00, 0.45) | 2.69 | 2.58 | 0.57 | -18.82* (-44.51, -1.68) |
| Muslim | 1997 | 2.22 | 4.35 | 1.57 | -2.48 (-5.31, 0.60) | 2.22 | 3.45 | 1.58 | -1.02 (-4.30, 2.76) |
| Muslim | 1998 | 3.05 | 4.24 | 1.78 | -2.48 (-5.31, 0.60) | 3.45 | 3.41 | 1.99 | -1.02 (-4.30, 2.76) |
| Muslim | 1999 | 1.93 | 4.13 | 1.41 | -2.48 (-5.31, 0.60) | 1.05 | 3.38 | 0.75 | -1.02 (-4.30, 2.76) |
| Muslim | 2000 | 6.40 | 4.03 | 2.67 | -2.48 (-5.31, 0.60) | 6.73 | 3.34 | 2.62 | -1.02 (-4.30, 2.76) |
| Muslim | 2001 | 5.92 | 3.93 | 2.68 | -2.48 (-5.31, 0.60) | 1.52 | 3.31 | 1.16 | -1.02 (-4.30, 2.76) |
| Muslim | 2002 | - | - | - | - | 2.12 | 3.27 | 1.52 | -1.02 (-4.30, 2.76) |
| Muslim | 2003 | 2.67 | 3.74 | 1.55 | -2.48 (-5.31, 0.60) | 2.36 | 3.24 | 1.42 | -1.02 (-4.30, 2.76) |
| Muslim | 2004 | 2.92 | 3.65 | 1.71 | -2.48 (-5.31, 0.60) | 2.87 | 3.21 | 1.75 | -1.02 (-4.30, 2.76) |
| Muslim | 2005 | 5.65 | 3.56 | 2.56 | -2.48 (-5.31, 0.60) | 3.56 | 3.17 | 1.84 | -1.02 (-4.30, 2.76) |
| Muslim | 2006 | 3.21 | 3.47 | 1.92 | -2.48 (-5.31, 0.60) | 4.28 | 3.14 | 1.80 | -1.02 (-4.30, 2.76) |
| Muslim | 2007 | 3.63 | 3.38 | 1.84 | -2.48 (-5.31, 0.60) | 1.65 | 3.11 | 1.17 | -1.02 (-4.30, 2.76) |
| Muslim | 2008 | 1.50 | 3.30 | 1.08 | -2.48 (-5.31, 0.60) | 4.29 | 3.08 | 2.03 | -1.02 (-4.30, 2.76) |
| Muslim | 2009 | 3.03 | 3.22 | 1.53 | -2.48 (-5.31, 0.60) | 1.89 | 3.04 | 1.38 | -1.02 (-4.30, 2.76) |
| Muslim | 2010 | 3.42 | 3.14 | 1.71 | -2.48 (-5.31, 0.60) | 4.40 | 3.01 | 2.06 | -1.02 (-4.30, 2.76) |
| Muslim | 2011 | 3.33 | 3.06 | 1.71 | -2.48 (-5.31, 0.60) | 2.93 | 2.98 | 1.23 | -1.02 (-4.30, 2.76) |
| Muslim | 2012 | 2.53 | 2.98 | 1.48 | -2.48 (-5.31, 0.60) | 4.51 | 2.95 | 1.92 | -1.02 (-4.30, 2.76) |
| Muslim | 2013 | 3.48 | 2.91 | 1.84 | -2.48 (-5.31, 0.60) | 0.92 | 2.92 | 0.53 | -1.02 (-4.30, 2.76) |
| Muslim | 2014 | 2.17 | 2.84 | 1.28 | -2.48 (-5.31, 0.60) | 2.12 | 2.89 | 1.01 | -1.02 (-4.30, 2.76) |
| Muslim | 2015 | 3.55 | 2.77 | 1.66 | -2.48 (-5.31, 0.60) | 2.98 | 2.86 | 1.41 | -1.02 (-4.30, 2.76) |
| Muslim | 2016 | 3.42 | 2.70 | 1.60 | -2.48 (-5.31, 0.60) | 3.85 | 2.83 | 1.56 | -1.02 (-4.30, 2.76) |
| Muslim | 2017 | 0.89 | 2.63 | 0.63 | -2.48 (-5.31, 0.60) | 3.79 | 2.8 | 1.57 | -1.02 (-4.30, 2.76) |
| Muslim | 2018 | - | - | - | - | 1.35 | 2.77 | 0.97 | -1.02 (-4.30, 2.76) |
| Muslim | 2019 | 1.71 | 2.50 | 1.21 | -2.48 (-5.31, 0.60) | 2.08 | 2.75 | 1.15 | -1.02 (-4.30, 2.76) |
| Muslim | 2020 | 2.94 | 2.44 | 1.38 | -2.48 (-5.31, 0.60) | 2.56 | 2.72 | 1.19 | -1.02 (-4.30, 2.76) |
| Buddhist | 1997 | 3.16 | 5.86 | 0.96 | -0.37 (-1.72, 21.93) | 3.00 | 3.49 | 0.80 | 1.75* (0.67, 4.87) |
| Buddhist | 1998 | 6.17 | 5.83 | 1.27 | -0.37 (-1.72, 21.93) | 4.17 | 3.55 | 0.93 | 1.75* (0.67, 4.87) |
| Buddhist | 1999 | 5.89 | 5.81 | 1.24 | -0.37 (-1.72, 21.93) | 2.79 | 3.61 | 0.76 | 1.75* (0.67, 4.87) |
| Buddhist | 2000 | 5.53 | 5.79 | 1.19 | -0.37 (-1.72, 21.93) | 4.41 | 3.67 | 0.92 | 1.75* (0.67, 4.87) |
| Buddhist | 2001 | 7.08 | 5.77 | 1.29 | -0.37 (-1.72, 21.93) | 4.69 | 3.74 | 1.00 | 1.75* (0.67, 4.87) |
| Buddhist | 2002 | 5.97 | 5.75 | 1.18 | -0.37 (-1.72, 21.93) | 4.16 | 3.80 | 0.88 | 1.75* (0.67, 4.87) |
| Buddhist | 2003 | 4.96 | 5.72 | 1.04 | -0.37 (-1.72, 21.93) | 4.24 | 3.87 | 0.89 | 1.75* (0.67, 4.87) |
| Buddhist | 2004 | 6.36 | 5.70 | 1.12 | -0.37 (-1.72, 21.93) | 4.43 | 3.94 | 0.89 | 1.75* (0.67, 4.87) |
| Buddhist | 2005 | 5.14 | 5.68 | 1.03 | -0.37 (-1.72, 21.93) | 2.45 | 4.01 | 0.61 | 1.75* (0.67, 4.87) |
| Buddhist | 2006 | 3.53 | 5.66 | 0.83 | -0.37 (-1.72, 21.93) | 4.15 | 4.08 | 0.79 | 1.75* (0.67, 4.87) |
| Buddhist | 2007 | 5.22 | 5.64 | 1.00 | -0.37 (-1.72, 21.93) | 3.65 | 4.15 | 0.77 | 1.75* (0.67, 4.87) |
| Buddhist | 2008 | 6.31 | 5.62 | 1.11 | -0.37 (-1.72, 21.93) | 3.94 | 4.22 | 0.74 | 1.75* (0.67, 4.87) |
| Buddhist | 2009 | 7.64 | 5.6 | 1.19 | -0.37 (-1.72, 21.93) | 4.40 | 4.29 | 0.83 | 1.75* (0.67, 4.87) |
| Buddhist | 2010 | 6.52 | 5.58 | 1.07 | -0.37 (-1.72, 21.93) | 3.38 | 4.37 | 0.66 | 1.75* (0.67, 4.87) |
| Buddhist | 2011 | 6.56 | 5.56 | 1.07 | -0.37 (-1.72, 21.93) | 4.54 | 4.45 | 0.79 | 1.75* (0.67, 4.87) |
| Buddhist | 2012 | 5.51 | 5.53 | 0.99 | -0.37 (-1.72, 21.93) | 3.27 | 4.52 | 0.65 | 1.75* (0.67, 4.87) |
| Buddhist | 2013 | 4.39 | 5.51 | 0.85 | -0.37 (-1.72, 21.93) | 4.21 | 4.60 | 0.76 | 1.75* (0.67, 4.87) |
| Buddhist | 2014 | 3.59 | 5.49 | 0.74 | -0.37 (-1.72, 21.93) | 4.70 | 4.68 | 0.75 | 1.75* (0.67, 4.87) |
| Buddhist | 2015 | 4.91 | 5.47 | 0.83 | -0.37 (-1.72, 21.93) | 5.47 | 4.77 | 0.82 | 1.75* (0.67, 4.87) |
| Buddhist | 2016 | 5.39 | 5.45 | 0.88 | -0.37 (-1.72, 21.93) | 5.67 | 4.85 | 0.80 | 1.75* (0.67, 4.87) |
| Buddhist | 2017 | 6.07 | 5.43 | 0.92 | joinpoint | 4.99 | 4.93 | 0.74 | joinpoint |
| Buddhist | 2018 | 3.98 | 4.43 | 0.74 | -18.46* (-42.11, -2.05) | 4.25 | 4.09 | 0.69 | -17.06* (-39.04, -2.18) |
| Buddhist | 2019 | 3.93 | 3.61 | 0.72 | -18.46* (-42.11, -2.05) | 3.14 | 3.39 | 0.56 | -17.06* (-39.04, -2.18) |
| Buddhist | 2020 | 2.87 | 2.94 | 0.57 | -18.46* (-42.11, -2.05) | 2.94 | 2.82 | 0.57 | -17.06* (-39.04, -2.18) |
