# Supplementary material for: Trends in skin cancer incidence in Songkhla, Southern Thailand, 1989–2020: A population-based study on the impact of geographic variation
Source: PLoS One. 2026 Jan 20;21(1):e0331635. doi: 10.1371/journal.pone.0331635 (PMC12818597; doi:10.1371/journal.pone.0331635)
Supplement: S3 Table — (DOCX) [file pone.0331635.s003.docx]

**S3 table.** Age effect of incidence rates (per 100,000) in men and women in Songkhla, Thailand, from 1989 to 2020, based on the Age-period-cohort analysis (AP-C and AC-P models)

| Sex | Men | | Women | |
| --- | --- | --- | --- | --- |
| Model | AP-C | AC-P | AP-C | AC-P |
| Age | Rate (95%CI) | Rate (95%CI) | Rate (95%CI) | Rate (95%CI) |
| 0-4 | - | - | 0.61 (0.31, 1.20) | 0.91 (0.31, 2.61) |
| 5-9 | 0.62 (0.33, 1.19) | 1.62 (0.57, 4.62) | - | - |
| 10-14 | 0.80 (0.45, 1.41) | 1.93 (0.76, 4.93) | 0.94 (0.55, 1.61) | 1.31 (0.55, 3.09) |
| 15-19 | 1.02 (0.62, 1.68) | 2.31 (1.01, 5.28) | 1.16 (0.72, 1.87) | 1.57 (0.73, 3.37) |
| 20-24 | 1.30 (0.84, 2.01) | 2.75 (1.34, 5.65) | 1.43 (0.95, 2.17) | 1.88 (0.96, 3.68) |
| 25-29 | 1.66 (1.14, 2.42) | 3.29 (1.78, 6.07) | 1.77 (1.24, 2.53) | 2.26 (1.26, 4.03) |
| 30-34 | 2.12 (1.54, 2.92) | 3.92 (2.35, 6.55) | 2.19 (1.62, 2.96) | 2.71 (1.66, 4.43) |
| 35-39 | 2.71 (2.06, 3.57) | 4.68 (3.08, 7.11) | 2.70 (2.09, 3.51) | 3.25 (2.15, 4.91) |
| 40-44 | 3.46 (2.71, 4.43) | 5.59 (3.99, 7.83) | 3.34 (2.65, 4.21) | 3.90 (2.77, 5.51) |
| 45-49 | 4.42 (3.48, 5.63) | 6.67 (5.03, 8.84) | 4.13 (3.31, 5.16) | 4.69 (3.48, 6.30) |
| 50-54 | 5.75 (4.47, 7.39) | 8.08 (6.24, 10.48) | 5.17 (4.10, 6.52) | 5.70 (4.32, 7.51) |
| 55-59 | 8.24 (6.49, 10.46) | 10.79 (8.60, 13.54) | 7.00 (5.54, 8.84) | 7.49 (5.80, 9.69) |
| 60-64 | 13.66 (10.83, 17.22) | 16.59 (13.67, 20.14) | 11.05 (8.90, 13.72) | 11.53 (9.38, 14.18) |
| 65-69 | 22.16 (17.51, 28.04) | 25.21 (20.98, 30.29) | 19.73 (15.55, 25.05) | 20.14 (16.50, 24.58) |
| 70-74 | 31.77 (24.72, 40.84) | 34.71 (28.22, 42.69) | 28.91 (23.14, 36.12) | 29.03 (24.05, 35.03) |
| 75-80 | 49.86 (39.20, 63.43) | 54.08 (43.35, 67.47) | 40.79 (32.57, 51.08) | 40.84 (32.79, 50.86) |
| 80-84 | 70.23 (55.15, 89.44) | 76.18 (59.71, 97.20) | 59.72 (46.80, 76.20) | 60.50 (46.33, 79.00) |
| 85+ | 89.57 (67.27, 119.27) | 97.18 (71.71, 131.71) | 78.21 (61.30, 99.80) | 80.75 (60.76, 107.32) |
