# Supplementary material for: Trends in skin cancer incidence in Songkhla, Southern Thailand, 1989–2020: A population-based study on the impact of geographic variation
Source: PLoS One. 2026 Jan 20;21(1):e0331635. doi: 10.1371/journal.pone.0331635 (PMC12818597; doi:10.1371/journal.pone.0331635)
Supplement: S4 Table — (DOCX) [file pone.0331635.s004.docx]

**S4 table.** Period effect of incidence rate ratios in men and women in Songkhla, Thailand, from 1985 to 2024, based on the Age-period-cohort analysis (AP-C and AC-P models)

| Sex | Men | | Women | |
| --- | --- | --- | --- | --- |
| Model | AP-C | AC-P | AP-C | AC-P |
| Calendar Year | RR (95%CI) | RR (95%CI) | RR (95%CI) | RR (95%CI) |
| 1985-1989 | 0.90 (0.80, 1.02) | 0.78 (0.57, 1.07) | 0.97 (0.86, 1.08) | 0.99 (0.74, 1.32) |
| 1990-1994 | 1.03 (1.00, 1.06) | 0.89 (0.75, 1.07) | 1.01 (0.98, 1.04) | 1.01 (0.85, 1.20) |
| 1995-1999 | 1.17 (0.97, 1.40) | 1.03 (0.91, 1.16) | 1.05 (0.89, 1.26) | 1.04 (0.92, 1.17) |
| 2000-2004 | 1.25 (0.95, 1.64) | 1.11 (0.97, 1.28) | 1.04 (0.80, 1.34) | 1.01 (0.88, 1.16) |
| 2005-2009 | 1.09 (0.85, 1.40) | 1.00 (0.87, 1.17) | 0.90 (0.71, 1.15) | 0.88 (0.75, 1.03) |
| 2010-2014 | 1.04 (0.81, 1.32) | 1.00 (0.88, 1.14) | 1.07 (0.85, 1.34) | 1.06 (0.94, 1.19) |
| 2015-2019 | 0.96 (0.76, 1.22) | 0.98 (0.87, 1.09) | 1.02 (0.82, 1.26) | 1.03 (0.93, 1.13) |
| 2020-2024 | 0.88 (0.63, 1.22) | 0.94 (0.73, 1.22) | 0.89 (0.66, 1.19) | 0.91 (0.73, 1.15) |
