# Supplementary material for: Trends in skin cancer incidence in Songkhla, Southern Thailand, 1989–2020: A population-based study on the impact of geographic variation
Source: PLoS One. 2026 Jan 20;21(1):e0331635. doi: 10.1371/journal.pone.0331635 (PMC12818597; doi:10.1371/journal.pone.0331635)
Supplement: S5 Table — (DOCX) [file pone.0331635.s005.docx]

**S5 table.** Cohort effect of incidence rate ratios in men and women in Songkhla, Thailand, from 1900 to 2009, based on the Age-period-cohort analysis (AP-C and AC-P models)

| Sex | Men | | Women | |
| --- | --- | --- | --- | --- |
| Model | AP-C | AC-P | AP-C | AC-P |
| Year of birth | RR (95%CI) | RR (95%CI) | RR (95%CI) | RR (95%CI) |
| 1900-1904 | 0.77 (0.51, 1.17) | 0.79 (0.48, 1.29) | 0.78 (0.48, 1.28) | 0.78 (0.44, 1.38) |
| 1905-1909 | 0.84 (0.62, 1.14) | 0.85 (0.58, 1.26) | 0.84 (0.59, 1.20) | 0.84 (0.54, 1.30) |
| 1910-1914 | 0.91 (0.75, 1.12) | 0.93 (0.69, 1.25) | 0.90 (0.71, 1.14) | 0.89 (0.64, 1.25) |
| 1915-1919 | 0.99 (0.87, 1.13) | 1.01 (0.79, 1.29) | 0.96 (0.82, 1.13) | 0.95 (0.73, 1.26) |
| 1920-1924 | 1.06 (0.92, 1.22) | 1.07 (0.84, 1.35) | 1.02 (0.87, 1.21) | 1.01 (0.77, 1.32) |
| 1925-1929 | 1.00 (0.89, 1.14) | 0.99 (0.79, 1.24) | 1.04 (0.90, 1.19) | 1.03 (0.81, 1.31) |
| 1930-1934 | 0.91 (0.76, 1.09) | 0.86 (0.66, 1.11) | 1.00 (0.85, 1.17) | 1.00 (0.78, 1.28) |
| 1935-1939 | 1.17 (0.99, 1.39) | 1.07 (0.89, 1.28) | 1.02 (0.88, 1.18) | 1.01 (0.79, 1.29) |
| 1940-1944 | 1.05 (0.88, 1.26) | 0.94 (0.87, 1.01) | 1.03 (0.90, 1.17) | 1.01 (0.89, 1.15) |
| 1945-1949 | 0.97 (0.85, 1.11) | 0.81* (0.68, 0.96) | 1.01 (0.85, 1.20) | 0.99 (0.96, 1.03) |
| 1950-1954 | 0.95 (0.81, 1.11) | 0.74* (0.56, 0.97) | 1.00 (0.88, 1.13) | 0.95 (0.79, 1.14) |
| 1955-1959 | 0.95 (0.81, 1.10) | 0.69* (0.51, 0.94) | 0.99 (0.85, 1.15) | 0.92 (0.69, 1.22) |
| 1960-1964 | 0.95 (0.82, 1.11) | 0.65* (0.46, 0.91) | 0.98 (0.85, 1.14) | 0.89 (0.64, 1.22) |
| 1965-1969 | 0.97 (0.81, 1.15) | 0.61* (0.41, 0.91) | 0.98 (0.85, 1.12) | 0.86 (0.61, 1.20) |
| 1970-1974 | 0.98 (0.77, 1.24) | 0.58* (0.35, 0.94) | 0.97 (0.83, 1.15) | 0.83 (0.57, 1.22) |
| 1975-1979 | 0.99 (0.72, 1.35) | 0.55* (0.30, 0.99) | 0.97 (0.78, 1.20) | 0.81 (0.51, 1.27) |
| 1980-1984 | 1.00 (0.68, 1.47) | 0.52 (0.25, 1.04) | 0.96 (0.73, 1.28) | 0.78 (0.46, 1.34) |
| 1985-1989 | 1.01 (0.63, 1.61) | 0.49 (0.21, 1.10) | 0.96 (0.67, 1.37) | 0.76 (0.40, 1.43) |
| 1990-1994 | 1.02 (0.59, 1.77) | 0.46 (0.18, 1.17) | 0.96 (0.62, 1.47) | 0.74 (0.36, 1.52) |
| 1995-1999 | 1.03 (0.55, 1.94) | 0.43 (0.15, 1.25) | 0.95 (0.58, 1.58) | 0.71 (0.31, 1.64) |
| 2000-2004 | - | - | 0.94 (0.49, 1.83) | 0.67 (0.24, 1.89) |
| 2005-2009 | - | - | 0.94 (0.45, 1.97) | 0.65 (0.21, 2.04) |
