# Supplementary material for: Trends in skin cancer incidence in Songkhla, Southern Thailand, 1989–2020: A population-based study on the impact of geographic variation
Source: PLoS One. 2026 Jan 20;21(1):e0331635. doi: 10.1371/journal.pone.0331635 (PMC12818597; doi:10.1371/journal.pone.0331635)
Supplement: Attachment — Submitted filename: Response_to_Reviewers_auresp_2.docx [file pone.0331635.s008.docx]

26/11/25

Emily Chenette

Editor-in-Chief

*PLOS ONE*

Dear Editor:

This letter accompanies the revised version of our manuscript entitled “Trends in Skin Cancer Incidence in Songkhla, Southern Thailand, 1989–2020: A Population-Based Study on the Impact of Geographic Variation” (PONE-D-25-44756), submitted for publication in PLOS ONE.

We appreciate the valuable comments and suggestions provided by the Associate Editor and the reviewers, which have greatly helped us improve the quality of our paper. All revisions have been marked using track changes.

Below, we provide a summary of the modifications made in the manuscript to address the reviewers’ comments.

We hope that our revisions and responses satisfactorily address all concerns and that the revised manuscript will be suitable for publication in PLOS ONE.

Sincerely,

Suchaya Pajareeyaphan, M.D.

**Point-by-point responses to reviewers**

**Journal Requirements:**

1. If the reviewer comments include a recommendation to cite specific previously published works, please review and evaluate these publications to determine whether they are relevant and should be cited. There is no requirement to cite these works unless the editor has indicated otherwise.

Response: The reviewer did not request citation of any specific articles, and no additional references were deemed necessary based on our evaluation. Therefore, no new citations have been added.

2. Please review your reference list to ensure that it is complete and correct. If you have cited papers that have been retracted, please include the rationale for doing so in the manuscript text, or remove these references and replace them with relevant current references. Any changes to the reference list should be mentioned in the rebuttal letter that accompanies your revised manuscript. If you need to cite a retracted article, indicate the article’s retracted status in the References list and also include a citation and full reference for the retraction notice.

Response: We thoroughly re-examined our reference list to ensure accuracy and completeness. All cited works were checked against PubMed and publisher databases for retraction status. None of the references in our manuscript were found to be retracted, and thus no removals or replacements were required. Accordingly, the reference list remains unchanged. We have ensured that all entries are formatted correctly according to the journal’s style and reflect the most up-to-date publication information.

**Reviewer #1**: Just want to recommend the author to enrich the conclusion, pointing out the impact of geographical and occupational factors in this part and what are the suggestions to prevent or manage the skin cancer based on the results of your study.

Response: We appreciate your suggestion. We have expanded and refined the conclusion to highlight the role of geographical and occupational factors and also added specific recommendations for prevention and management of the skin cancer. These revisions improve the clarity and practical relevance of the conclusion and are now reflected in the revised manuscript (Conclusion section, page 11, lines 10-16).
